# Two male-killing *Wolbachia* from *Drosophila birauraia* that are closely related but distinct in genome structure

**DOI:** 10.1098/rsos.231502

**Published:** 2024-01-10

**Authors:** Hiroshi Arai, Masayoshi Watada, Daisuke Kageyama

**Affiliations:** ^1^ National Agriculture and Food Research Organization (NARO), 1-2 Owashi, Tsukuba, Ibaraki 305-0851, Japan; ^2^ Graduate School of Science and Engineering, Ehime University, Matsuyama, Ehime 780-8857, Japan; ^3^ Department of Biological Sciences, Tokyo Metropolitan University, 1-1 Minamiosawa, Hachioji, Tokyo 192-0397, Japan

**Keywords:** male killing, evolution, *Wolbachia*, genome rearrangement

## Abstract

Insects harbour diverse maternally inherited bacteria and viruses, some of which have evolved to kill the male progeny of their hosts (male killing: MK). The fly species *Drosophila biauraria* carries a maternally transmitted MK-inducing partiti-like virus, but it was unknown if it carries other MK-inducing endosymbionts. Here, we identified two male-killing *Wolbachia* strains (*w*Biau1 and *w*Biau2) from *D. biauraria* and compared their genomes to elucidate their evolutionary processes. The two strains were genetically closely related but had exceptionally different genome structures with considerable rearrangements compared with combinations of other *Wolbachia* strains. Despite substantial changes in the genome structure, the two *Wolbachia* strains did not experience gene losses that would disrupt the male-killing expression or persistence in the host population. The two *Wolbachia*-infected matrilines carried distinct mitochondrial haplotypes, suggesting that *w*Biau1 and *w*Biau2 have invaded *D. biauraria* independently and undergone considerable genome changes owing to unknown selective pressures in evolutionary history. This study demonstrated the presence of three male-killers from two distinct origins in one fly species and highlighted the diverse and rapid genome evolution of MK *Wolbachia* in the host.

## 1. Background

Insects often harbour endosymbiotic microbes that are transmitted from the female host to the offspring [1]. Males are an evolutionary dead-end for maternally transmitted microbes, and the lack of transmission through male hosts often leads to the evolution of reproductively parasitic traits [1–3]. In some cases, this is manifested in the evolution of male-killing (MK) phenotypes, in which male offspring of infected mothers are killed during development [1–4]. MK is thought to be advantageous for the maternally transmitted microbes, and indeed various microbes such as bacteria, microsporidia and viruses induce MK in respective insect species [3,4]. In addition, multiple male killers sometimes infect the same host species [5,6].

The endosymbiotic bacterium, *Wolbachia* (Alphaproteobacteria), is present in at least 40% of all insect species, making it one of the most widespread endosymbionts [7–9]. *Wolbachia* is maternally transmitted but is considered to have experienced host shifts repeatedly in its evolutionary history. The high prevalence of *Wolbachia* in arthropods is partly due to *Wolbachia*-induced host manipulations, such as cytoplasmic incompatibility (CI), parthenogenesis, feminization and MK [7,8,10]. *Wolbachia* induce MK in particularly diverse host species, however, how *Wolbachia* acquired and maintained their MK abilities on an evolutionary timescale remains largely unknown [11–13].

Genomic changes that lead to phenotypic changes and adaptation to new hosts are critical for the evolution of *Wolbachia* [14–18]. For example, the MK *Wolbachia* strain, *w*Hm-t, is thought to have evolved from its closely related non-MK strain, *w*Hm-c, in the tea tortrix, *Homona magnanima*, by acquiring an MK-associated prophage region [18]. By contrast, *Ostrinia furnacalis* and *Ostrinia scapulalis* harbour closely related MK *Wolbachia* strains that show an extremely high degree of genomic similarity to several inversions [19]. The MK *Wolbachia* strains in *Ostrinia* moths are thought to have descended from their common ancestral hosts and have maintained a stable genome structure throughout their evolution [19]. In the highly host-dependent symbiotic bacterium, *Wolbachia*, substantial changes in the genome structure can disrupt phenotypic expression and intergenic interactions, posing major risks to *Wolbachia* fitness. However, large-scale genome changes can also drive bacterial evolution because they can alter gene expression and phenotypic outcomes in ways that point mutations cannot [20,21]. The *Wolbachia* genome is an intriguing subject for exploring the evolutionary interactions between facultative endosymbionts and their hosts.

In this study, we identified two novel, closely related MK *Wolbachia* strains (*w*Biau1 and *w*Biau2) infecting *Drosophila biauraria*. We analysed and compared their genomes with other *Wolbachia* strains identified from diverse insects to elucidate the evolutionary history of the MK *Wolbachia* strains. Further, we compared their genomes with the partiti-like virus DbMKPV1, which induces MK during the late embryonic stage (i.e. early MK) in *D. biauraria* [22,23], to clarify the evolutionary origin of MK in this fly species.

## 2. Methods

### 2.1. Collection and rearing of *Drosophila biauraria*

*Drosophila biauraria* samples were collected from the Field Science Center for Northern Biosphere, Hokkaido University, Tomakomai, Hokkaido, Japan in 2015 and 2017. Flies were collected by sweeping and banana traps. The collected females were individually maintained at 19°C with the standard banana medium [22]. The sex ratios of the lines derived from field-collected females were determined at the adult stage. The normal sex ratio (NSR) isofemale line SP11-20 [23] was maintained for more than 70 generations. The all-female matrilines (W1 and W2), each derived from a single female, were maintained by crossing with males of the SP11-20 line. *Wolbachia* and the MK partiti-like virus DbMKPV1 infections were detected by PCR, as described previously [22,23].

### 2.2. Tetracycline treatment

All-female matrilines (W1 and W2) were reared on tetracycline-containing banana medium (0.05% [w/v]) [22] for two generations.

### 2.3. Egg-hatching rates

Egg-hatching rates were estimated by counting the number of hatched and unhatched larvae. A total of 50–100 females of either W1 or NSR (SP11-20) were allowed to oviposit on grape juice agar medium for 1 day [23]. The eggs were collected and maintained in phosphate-buffered saline with Tween 20 (PBST; 137 mmol l^−1^ NaCl, 8.1 mmol l^−1^ Na_2_HPO_4_, 2.68 mmol l^−1^ KCl, 1.47 mmol l^−1^ KH_2_PO_4_, 0.02% Tween 20, pH7.4) for 4 days. The number of hatched larvae and remaining embryos were counted manually under a microscope. This treatment was repeated at least four times.

### 2.4. Sex determination of embryos and hatchlings of *D. biauraria*

We determined the sex of embryos and hatchlings by PCR targeting a male-specific Y chromosome marker. Briefly, each embryo and hatched larvae was squashed in 20 μl of PrepMan Ultra Sample Preparation Reagent (ThermoFisher). Samples were then incubated at 100°C for 10 min, vortexed for 15 s, centrifuged at 20 000 × *g* for 2 min, and finally subjected to PCR. A Y chromosome-linked male-specific marker for *D. biauraria* [24] was amplified using a pair of primers, DbY_c52202_F2 (5′-ACCGAGCGCGAAATCATAAAACCAGCATC-3′) and DbY_c52202_R2 (5′-CTCATATCACTTCATGTATCCCACACTTTTAACAG-3′). Db-actin5C-68-F (5′-GGCCATCCAGGCCGTGCTCTC-3′) and Db-actin5C-68-R (5′-GCGCTCGGCAGTGGTGGTGAAG-3′) were used to amplify *actin-5C* to confirm proper *D. biauraria* genomic DNA extraction. These markers were amplified using the Emerald Amp Max Master mix (TaKaRa) at 94°C for 3 min; the cycling conditions were as follows: 35 cycles of denaturation at 94°C for 30 s, annealing at 55°C for 30 s, and extension at 72°C for 30 s, followed by a final extension at 72°C for 7 min. Of the actin-positive samples, those that were positive for Y-markers were classified as male. Those that were negative for Y-markers were classified as female.

### 2.5. Genome sequencing of flies and constructions of *Wolbachia* genomes

For genome sequencing of fly lines W1 and W2, high molecular weight DNA was extracted from 0.1 g adult females (approximately 100–200 individuals) by using Nanobind Tissue Big DNA Kit (Circulomics Inc., Baltimore, MD, USA) and was used for library construction using Ligation Sequencing Kit v14 (Oxford Nanopore Technologies, Oxford, UK) following the manufacturer's protocol. The constructed libraries were sequenced using the ONT MinION flow cell (R 10.4) (Oxford Nanopore Technologies). The extracted DNA was also subjected to Illumina paired-end 150 bp sequencing (PE-150) at the Bioengineering Lab. Co., Ltd. (Japan). The obtained nanopore reads were assembled using Flye 2.3 [25] in Galaxy Europe (https://usegalaxy.eu/). Homologies between the assembled contigs of W1 and W2 and all *Wolbachia* genomes available in the NCBI database were assessed using BLASTn searches. Contigs showing homology to known *Wolbachia* genomes were designated as candidate contigs of *Wolbachia* strains in *D. biauraria*. The raw data of W1 and W2 were mapped to *Wolbachia*-like contigs using minimap2 v2.17-r941 [24], and the mapped reads were extracted using SAMtools v.1.9 [26] and assembled using Flye 2.3 [24]. The circularity of the *Wolbachia* wBiau1 and wBiau2 genomes was confirmed using Bandage v0.8.1 [27]. Circular *Wolbachia* genomes were polished against Illumina data using minimap2 [24] and Pilon v. 1.23 [28]. The polished closed genomes of the wBiau1 and wBiau2 strains were annotated via the DFAST web server [29]. Prophage regions were annotated using the PHASTER web server [30]. Insertion sequence (IS) elements in *Wolbachia* genomes were further annotated using ISEScan [31].

*Wolbachia* genes *wmk* [32], *cifs (cifA and cifB)* [32–34], and *oscar* [18,35] were used to identify homologues in the *w*Biau1 and *w*Biau2 genomes using local BLASTn and BLASTp searches (default parameters). Motifs in the *wmk*, *cifA, cifB,* and *oscar* gene homologues were surveyed using InterPro (https://www.ebi.ac.uk/interpro/) and HHpred (https://toolkit.tuebingen.mpg.de/tools/hhpred). Phylogenetic trees of *Wolbachia wsp* and *MLST* genes were constructed based on maximum likelihood with bootstrap re-sampling of 1,000 replicates using MEGA7 [36].

### 2.6. Phylogenetic analysis of mitochondrial CO1

The mitochondrial CO1 of *D. biauraria* lines was amplified using HCO and LCO primer sets targeting the CO1 gene [37]. Amplicons were purified with Wizard SV Gel and PCR Clean-Up System (Promega), which were subjected to sequencing using BigDye terminator v3.1 (Applied Biosystems) with the following conditions: 96°C for 1 min, followed by 25 cycles of 96°C for 10 s, 50°C for 5 s, and 60°C for 4 min. A phylogenetic tree of *CO1* was constructed based on maximum likelihood with bootstrap re-sampling of 1,000 replicates using MEGA7 [36].

### 2.7. Statistical analysis

The sex ratio of the adult flies was assessed using Fisher's exact test. Egg hatching rates were analysed using the Wilcoxon test. All analyses were performed using the R software v4.0 (https://www.r-project.org/). *P* values <0.05 were considered significant.

### 2.8. Data accessibility

The sequence read data were deposited in the DDBJ under the accession numbers PRJDB16258 (BioProject), SAMD00634859–SAMD00634860 (BioSample), and DRA016759 (DRA). *Wolbachia* genomes are available in the DDBJ database under the accession numbers *w*Biau1 (AP028655) and *w*Biau2 (AP028656). Any additional information required to reanalyse the data reported in this paper is accessible from the Dryad Digital Repository: https://doi.org/10.5061/dryad.j9kd51cjh [38].

## 3. Results and discussion

### 3.1. *Wolbachia* induces MK in *Drosophila biauraria*

**Figure 1.**
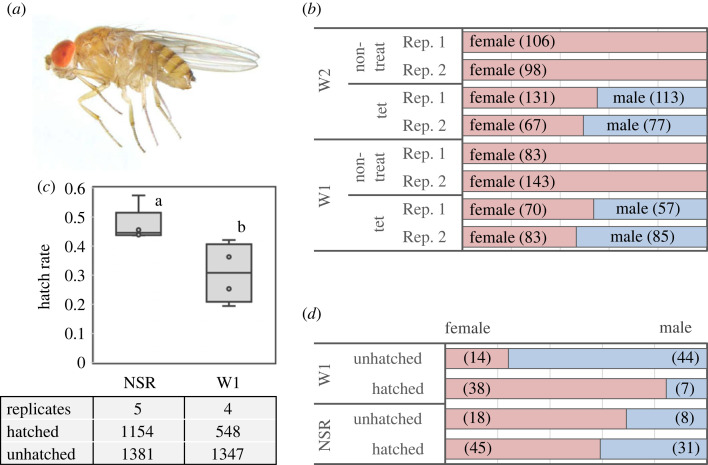
Sex ratio distortion in *Drosophila biauraria* matrilines. (*a*) Morphology of *D. biaurara* female adult (W1 line). (*b*) Sex ratio of adult flies in *Wolbachia*-infected W1 and W2 matrilines with and without tetracycline treatment. Each treatment had two replicates. Sample sizes (number of individuals examined) are given in parentheses. (*c*) Hatchability of W1 and uninfected matrilines (NSR, SP11-20). The total number of replicates, hatched and unhatched individuals, are shown below the whisker plot. The horizontal bar within the box represents the median. The upper and lower hinges of the box indicate the upper and lower quartiles, respectively. Different letters indicate significant differences between groups (Wilcoxon test, *p* < 0.05). (*d*) Sex ratio of hatched larvae and unhatched embryos examined by PCR assays targeting the male-specific Y chromosome. Sample sizes (number of examined individuals) are given in parentheses. NSR, normal sex ratio.

We collected 124 matrilines of *D. biauraria* (figure 1*a*) from Tomakomai, Hokkaido, in 2015 (*n* = 55) and 2017 (*n* = 69). Of these, 118 matrilines showed normal sex ratios (approximately 1:1, male: female), whereas six matrilines were all female. Six all-female matrilines were negative for DbMKPV1 [23], but positive for *Wolbachia* (table 1). Males were restored by tetracycline treatment, which was performed on two *Wolbachia*-infected all-female matrilines, TM15-28 (referred to as W1) and TM17-F3 (referred to as W2) (figure 1*b*). The egg hatching rates examined for W1 were significantly lower (28.9%, five replicates, *n* = 2535 in total) than those of the normal sex ratio line SP11-20 (45.5%, four replicates, *n* = 1895 in total) (Wilcoxon test, *p* = 0.01, figure 1*c*). The low hatching rates in both lines may partly be explained by the high rate of unfertilized eggs derived from inbreeding depression. Furthermore, sex-determination based on PCR detection of the male-specific Y chromosome in the W1 matriline showed that sex was significantly male-biased in unhatched embryos (75.8% male: 44 males and 14 females, binomial test, *p* = 0.0002, figure 1*d*) and significantly female-biased in hatched larvae (84.4% female: 7 males and 38 females, binomial test, *p* = 0.004). Exceptionally, some adult males emerged from a few vials during fly maintenance, but none emerged during our experiment. Thus, the *Wolbachia*-infected male hatchlings appear to die before reaching the adult stage. Similarly, in the DbMKPV1-infected *D. biauraria* strain (although no adult males were ever observed within it), a few male larvae occasionally hatch but die before reaching adulthood [23]. These results suggest that MK occurs primarily during embryonic development (early MK), but the effect of MK is continuously active at later stages of *D. biauraria*.

**Table 1.** Frequencies of all-female lines among iso-female lines established for *Drosophila biauraria.*

year	all-female lines		normal sex ratio lines	total
	*Wolbachia*	DbMKPV1		
2015	3	0	52	55
2017	3	0	66	69

### 3.2. *Wolbachia* strains *w*Biau1 and *w*Biau2 are closely related but have a high level of genome rearrangements

**Figure 2.**
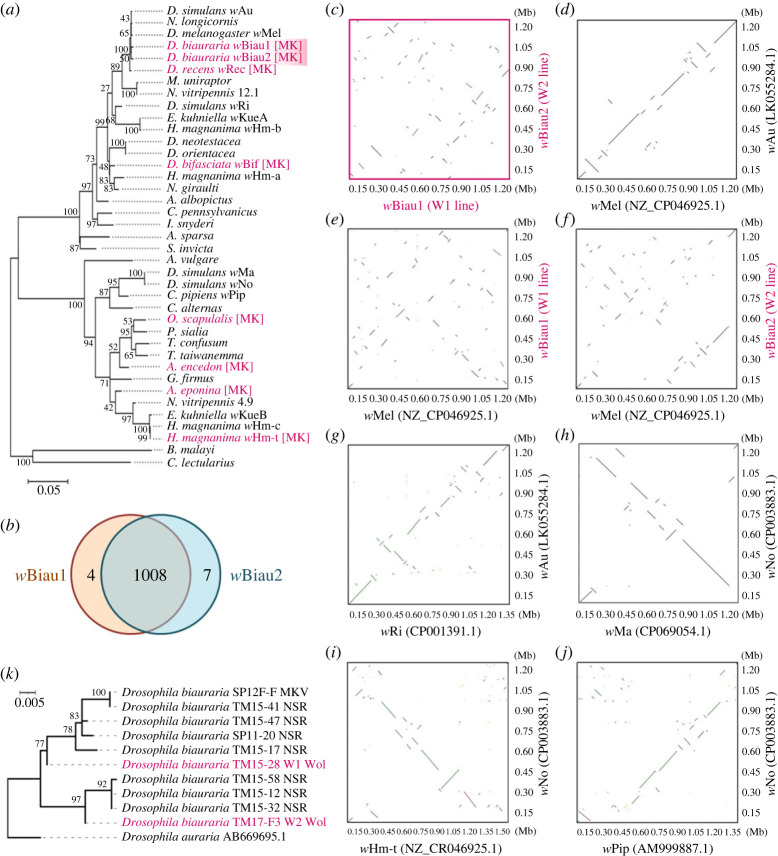
Genomic similarities between *Wolbachia* strains *w*Biau1 and *w*Biau2 and phylogeny of the host *D. biauraria*. (*a*) Phylogenetic tree of concatenated *Wolbachia wsp* and *MLST* sequences constructed based on maximum likelihood with bootstrap re-sampling of 1,000 replicates using MEGA7 [37]. Homologs of *Wolbachia* strains were quoted from the *Wolbachia MLST* database. (*b*) Protein clusters conserved within *w*Biau1 and *w*Biau2 strains. (*c–j*) Dot plots showing conserved syntenies between *Wolbachia* strains. *w*Biau1 and *w*Biau2 in *D. biauraria* (*c*), *w*Mel and *w*Au in *D. melanogaster* and *D. simulans,* respectively (*d*), *w*Biau1 and *w*Mel (*e*), *w*Biau2 and *w*Mel (*f*), *w*Au and *w*Ri in *D. simulans* (*g*), *w*Ma and *w*No in *D. simulans* (*h*), *w*Hm-t in *H. magnanima* and *w*No (*i*), and *w*Pip in *C. pipiens* and *w*No (*i*), were compared. (*k*) Phylogenetic tree of mitochondria COI sequences of *D. biauraria. Drosophila auraria* [GenBank AB669695.1] was used as an outgroup.

Genome sequencing of W1 and W2 flies using both the Nanopore and Illumina platforms generated circular closed *Wolbachia* genomes (table 2). Other than *Wolbachia*, no known MK bacteria (*Spiroplasma*, *Rickettsia*, *Cardinium* and *Arsenophonus*) or microsporidia were identified from the genome read data, and all the bacterial reads were considered to be derived from gut symbionts or environmental bacteria (electronic supplementary material, table S1). This suggests that *Wolbachia* is the cause of MK in both W1 and W2 lines. The W1 and W2 matrilineal *Wolbachia* were closely related but had different nucleotide sequences in *Wolbachia* typing genes (*wsp* and *MLST*) (figure 2*a*). Therefore, we designated the *Wolbachia* strains as *w*Biau1 (W1 line) and *w*Biau2 (W2 line). Both *w*Biau1 (1178058 bp circular genome) and *w*Biau2 (1183391 bp circular genome) belonged to supergroup A-type *Wolbachia* (figure 2*a* and table 2) and shared most genes with high similarity (figure 2*b* and electronic supplementary material, table S2). Despite their high similarity in genetic components, *w*Biau1 and *w*Biau2 showed a high degree of genome rearrangement (i.e. many genomic shifts and inversions) (figure 2*c*). This high level of genomic rearrangement was not observed between the closely related supergroup A *Wolbachia* strains: *w*Mel in *Drosophila melanogaster* (1,267,783 bp, NZ_CP046925.1) and *w*Au in *Drosophila simulans* (1,268,461 bp, LK055284.1; figure 2*d*). Compared with *w*Mel, both *w*Biau1 and *w*Biau2 showed high levels of genomic rearrangement (figure 2*e*,*f*). This was further supported by the comparisons of the *w*Biau strains with *w*Ri from *D. simulans* (1,445,873 bp, CP001391.1), which is more distantly related to *w*Mel (figure 2*a* and electronic supplementary material, figure S1). In supergroup A, even moderately divergent strains *w*Au and *w*Ri in the identical host, *D. simulans* showed a certain degree of synteny (figure 2*g*). In supergroup B, the closely related strains *w*Ma (1,273,535 bp, CP069054.1) and *w*No (1,301,823 bp, CP003883.1) in *D. simulans* also showed a certain degree of synteny (figure 2*h*). Furthermore, the distantly related *Wolbachia* strains, *w*Hm-t (1,542,158 bp, AP025638) in *Homona magnanima* and *w*Pip (1,482,455 bp, AM999887.1) in *Culex pipiens* shared more collinear genomes with the *w*No strain (figure 2*i*,*j*) than with the combination of *w*Biau1 and *w*Biau2. The *Wolbachia* strains *w*Biau1 and *w*Biau2 harbour two and three prophage candidate regions, respectively (electronic supplementary material, figure S2). In addition, *w*Biau1 encoded 88 IS elements in its genome (covering 10.1% of the genome), and *w*Biau2 encoded 72 IS elements (7.74%), which were similar to other *Wolbachia* strains (e.g. *w*Mel: *n* = 69, 7.05%; *w*Au: *n* = 71; 7.60%; wRi: *n* = 128; 12.3%; wHm-t: *n* = 136; 11.2%) (electronic supplementary material, table S3).

**Table 2.** General characteristics of *Wolbachia* genomes. GC, guanine-cytosine; CDS, coding sequences; tRNA, transfer RNA; rRNA, ribosomal RNA; MK, male killing.

strain	*w*Biau1	*w*Biau2
supergroup	A	A
phenotype	MK	MK
contigs	1	1
genome length (nt)	1,178,058	1,183,391
GC content (%)	35.0	35.2
CDS	1251	1,233
tRNA	34	34
rRNA	3	3

Bacterial chromosomes are dynamic structures shaped by long evolutionary histories [21,39,40]. Compared with a free-living lifestyle, a host-restricted lifestyle may impose different selective forces on endosymbiont genome evolution [40]. Endosymbiotic bacteria typically exhibit highly reduced AT-rich genomes acquired through a combination of genomic rearrangements and the accumulation of nucleotide substitutions/deletions [40–44]. This is thought to be a consequence of the intracellular lifestyle of endosymbionts, in which bacteria experience severe bottlenecks during host reproduction, leading to reduced selection against deleterious mutations. The degradation process is further facilitated by the stability of the nutrient-rich cellular environment in which endosymbiotic bacteria reside, allowing gene loss without reducing the fitness of endosymbionts during long coevolutionary relationships [45,46]. For example, the endosymbiotic bacterium, *Buchnera,* shows many chromosomal rearrangements and deletions compared with its free-living enteric relatives, suggesting that the early stages of its evolutionary transition to a host-restricted lifestyle are highly dynamic [47]. However, the *Buchnera* genome structure has been extraordinarily stable over the past 100 million years of diversification between aphid species, despite high levels of divergence in gene sequence [48,49]. *Wolbachia* strains have relatively stable genome structures, although they are more dynamic than *Buchnera* strains [18,50,51]. In this study, we could not identify plausible features that could have driven the genome diversity of the *w*Biau strains (cf. the number of transposable elements such as prophages and IS elements were comparable to other *Wolbachia* strains), but there may be some unknown mechanism that allows exceptionally high rates of genome arrangement in *w*Biau1 and *w*Biau2. Despite the substantial and rapid changes in the genome structure, it appears that the *w*Biau strains did not experience gene loss that would eliminate MK expression or persistence in the host population.

### 3.3. *w*Biau1 and *w*Biau2 do not encode the MK gene of DbMKPV1

To clarify whether the *Wolbachia* strains encoded the MK gene of the Partiti-like virus DbMKPV1 in *D. biauraria* [23], we assessed the homology of their genes using a BLAST search, which revealed that they did not contain any genes with high homology (electronic supplementary material, table S4). As suggested by *Homona* moth, which harbours three different male killers (*Wolbachia*, *Spiroplasma* and a Partiti-like virus OGV) [18,52,53], MK *Wolbachia* and DbMKPV1 are likely to induce MK in *D. biauraria* via different mechanisms (causative genes) and have acquired these mechanisms independently through different evolutionary processes.

### 3.4. *w*Biau1 and *w*Biau2 harboured MK- and CI-associated genes of *Wolbachia*

We found that *w*Biau1 and *w*Biau2 encoded one (WBIAU1_10910 [154 aa]) and two (WBIAU2_11720 [123 aa] and WBIAU2_11740 [74 aa]) *wmk* homologues encoding single helix-turn-helix (HTH) domain, respectively (BLASTp searches, electronic supplementary material, table S4). Of these, WBIAU1_10910 and WBIAU2_11720 genes showed high homology (identity: 99.2%, e-value: 0, bit score: 671, BLASTn, electronic supplementary material, table S5), but no homologue of WBIAU2_11740 was identified in the genome of *w*Biau1. The adjacent gene of the *wmk* homologues (*w*Biau1: WBIAU1_10920 [298 aa]; *w*Biau2: WBIAU2_11710 [298 aa] and WBIAU2_11730 [53 aa]) also showed partial homology to the *wmk* of *w*Mel (electronic supplementary material, table S4), but the proteins encoded by the genes lacked the HTH domain. Some *wmk* genes are known to induce male lethality in *Drosophila melanogaster* [18,33]. In addition, some tandemly arrayed *wmk* homologues show combined actions to induce male lethality when overexpressed in *D. melanogaster* [32]. While the *wmk* homologues found in the *w*Biau strains were relatively smaller than that found in *w*Mel (302 aa), the adjacent *wmk* homologues may be involved in MK in *D. biauraria*. By contrast, neither *w*Biau1 nor *w*Biau2 carry the *oscar* gene, which induces MK in *Ostrinia* moths, where the Oscar protein degrades the host's male-determining factor, *masculinizer* (*masc*) [35]. Interestingly, Oscar is not conserved among MK *Wolbachia* strains and does not function in *D. melanogaster* which lacks the *masc* gene [18]. Thus, MK mechanisms (causative genes) in *D. biauraria* are likely to be different from those in *Ostrinia* moths.

We also found that both *w*Biau1 and *w*Biau2 harboured adjacent *cif* genes, *cifA* (WBIAU1_10970 [491 aa] and WBIAU2_11670 [474 aa]) and *cifB* (WBIAU1_10980 [1179 aa] and WBIAU2_11660 [1173 aa]) (electronic supplementary material, figure S3 and table S4). The *w*Biau strains showed very high homology in the *cifA* (identity: 98.5%, e-value: 0, bit score: 2372, BLASTn) and *cifB* (identity: 96.7%, e-value: 0, bit score: 5856) genes between the strains (electronic supplementary material, table S5). The CifB proteins of both *Wolbachia* strains encoded a deubiquitinase domain and were classified as the type I CifB as found in the *w*Mel strain [32]. The *cif* genes (CifA and CifB) are causative factors of *Wolbachia*-induced CI, in which the offspring of infected males and uninfected females are lethal during development [32,34]. This phenotype allows *Wolbachia* to spread rapidly throughout the host populations [11]. However, the MK phenotype induced by *w*Biau1 and *w*Biau2 does not allow CI expression, which requires infected males. Note that the production of infected males by the nuclear suppressors against MK should result in CI expression [54]. However, suppressors against MK are not selectively favoured when the prevalence of MK microbe is low [55], as in the case of *D. biauraria* (table 1). Therefore, the potential CI ability possessed by *w*Biau1 and *w*Biau2 is not selectively favoured. The fact that the *cif* genes remained intact despite substantial genome rearrangements may suggest that *cif*s have unknown pleiotropic functions in addition to CI that are adaptive for *Wolbachia* (e.g. host protective phenotypes or MK).

### 3.5. Evolutionary history of *Wolbachia* infection in *D. biauraria*

The mitochondrial CO1 sequences of MK lines TM15-28 (W1), TM17-F3 (W2) and SP12F), were distinct (figure 2*k*). While the mitochondrial haplotype of DbMKPV1-infected SP12F was identical to that of the normal sex ratio line TM15-41, the haplotypes of the *Wolbachia*-infected W1 and W2 differed from those of the DbMKPV1-infected and uninfected hosts and were located at the basal branches in the two distinct clades. Although we were unable to assess the *Wolbachia* genomes and mitotypes of the other four MK matrilines collected in 2015 and 2017 (table 1) due to the loss of fly stocks during laboratory maintenance, our data suggest that the invasion of *w*Biau1 and *w*Biau2 occurred earlier than the divergence of the two clades.

## 4. Conclusion

In summary, our study highlights the diverse and rapid evolution of the MK *Wolbachia* genome through its interactions with host insects. The two *Wolbachia*-infected matrilines of *D. biauraria* carried distinct mitochondrial haplotypes; therefore, we postulate that the evolutionary history of the MK *Wolbachia* genome is shaped by the independent invasion of *D. biauraria* by *w*Biau1 and *w*Biau2.

This study is limited in that it could not establish the selective pressures that have driven *Wolbachia* genome changes. Further comparative genomics of closely related *Wolbachia* strains in a host species and experimental evolutionary assays, such as studying genome changes after *Wolbachia* transfer into a new host, may shed light on their genome evolution dynamics as well as the evolutionary interactions between *Wolbachia* and host insects.

## Data Availability

The sequence read data were deposited in the DDBJ under the accession numbers PRJDB16258 (BioProject), SAMD00634859–SAMD00634860 (BioSample), and DRA016759 (DRA). Wolbachia genomes are available in the DDBJ database under the accession numbers wBiau1 (AP028655) and wBiau2 (AP028656). Any additional information required to reanalyse the data reported in this paper is accessible from the Dryad Digital Repository: https://doi.org/10.5061/dryad.j9kd51cjh [38]. Supplementary material is available online [56].
